# Seasonal Spatial Distribution Patterns and Climate Scenario Predictions of *Palaemon gravieri*: A Key Shrimp Species Depressing Jellyfish Blooms in the East China Sea Region

**DOI:** 10.3390/biology14081095

**Published:** 2025-08-21

**Authors:** Min Xu, Jianzhong Ling, Haisu Zheng, Xiaojing Song, Huiyu Li

**Affiliations:** 1 Key Laboratory of Fisheries Remote Sensing Ministry of Agriculture and Rural Affairs, East China Sea Fisheries Research Institute, Chinese Academy of Fishery Sciences, Shanghai 200090, China; xumin@ecsf.ac.cn (M.X.); lingjz@ecsf.ac.cn (J.L.); songxiaojing@ecsf.ac.cn (X.S.); 2 Shanghai Aquatic Wildlife Conservation and Research Center, Shanghai 200080, China; zhenghaisu322@hotmail.com

**Keywords:** climate change, Dasha, Palaemonidae, jellyfish bloom, Sand Crab, stock assessment, total allowable catch, Yangtze River mouth

## Abstract

*Palaemon gravieri* is an ecologically important shrimp species that plays a vital role in depressing jellyfish blooms in the southern Yellow and East China Seas of China. However, information on its distribution pattern and migration route related to environmental variables is fragmented. We conducted independent trawling surveys of *P. gravieri* between 2018 and 2019. Its sea surface temperature and sea surface salinity lower limits were 8 °C and 30‰, respectively. It showed the highest abundance at sea bottom temperatures and salinities of 12–14 °C and 32–33‰, respectively, in spring; 11–12 °C and 32.5‰ in autumn; and 10.5–14 °C and 31–33‰ in winter. Mean biomass, abundance, and size were ranked seasonally as follows: autumn > winter > spring > summer; autumn > winter and spring; and summer > spring > autumn > winter, respectively. Under the current climate scenario, *P. gravieri* is mainly concentrated in the inshore areas of the southern Yellow and northern East China Seas. Under SSP1-2.6, SSP2-4.5, SSP3-7.0, and SSP5-8.5 scenarios in 2100, *P. gravieri* was mainly concentrated in the southern Yellow and northern East China Seas, and in inshore areas of the East China Sea. This species is therefore expected to benefit from climate warming. The findings of this study can facilitate the development of climate-induced fishery strategies and management schemes.

## Simple Summary

Sustainable fisheries management schemes need to incorporate numerous ecological aspects of the species, such as seasonal and spatial distribution patterns and responses to environmental and climate pressures. The shrimp *Palaemon gravieri* is an ecologically important species in the southern Yellow and East China Seas. It has a very high nutritional value and is a potentially important industrial aquacultural species in both China and Korea. It also preys on the scyphistomae of jellyfish *Aurelia aurita*, thus having an important role in suppressing jellyfish blooms. However, information on its distribution patterns and migration is fragmented. This study revealed that its recruitment is mostly concentrated in longitudes 123° E–124° E. Furthermore it predominantly spawns in the Yangtze River mouth fishing ground in spring, and has overwintering and nursery grounds in the Dasha fishing ground. These findings can benefit the formulation of total allowable catch fisheries management.

## 1. Introduction

The shrimp *Palaemon gravieri* (Arthropoda, Malacostraca, Decapoda, Palaemonidae, Palaemon), commonly known as ‘Hongmangzi’ and ‘Taohongxia’, is endemic to the Northwest Pacific Ocean, from the north of Taiwan to the west coast of North Korea [1]. It is a fast-growing species with a body length of 50 to 70 mm [2], also working as a potential industrial aquacultural shrimp species [3]. Morphologically, it has a relatively short body shape, slender feet, and a light-yellow body color with brownish-red stripes. It is an ecologically important species, playing a key role in controlling jellyfish blooms in the East China Sea region (including the southern Yellow and East China Seas) by preying on the scyphistomae of *Aurelia aurita* [1]. It is also a potential industrial aquacultural shrimp species [3].

In the Bohai, Yellow, and East China Seas, *P. gravieri* dominates throughout the year and it is the economically important species among the Palaemonidae family [4]. Its total catch accounts for 12.59% of the total shrimp catch in China, with an annual output of 46,000 t [5]. Furthermore, it comprises the raw material of the famous seafood ‘Huanglongxiami’ in the Shengsi Islands of China [4].

Regarding its nutritional content, the protein and fat percentage is 20% higher and 40% lower, respectively, than that of lean pork and river prawn, and it contains more than 40% vitamin A and other trace elements, including vitamin E, iodine, and phosphatide [6]. In autumn and winter, *P. gravieri* comprises 8–9% phospholipids, representing 34–36% of the total lipids [7].

*P. gravieri* catches in the southern Yellow and East China Seas slightly increased between the 1980s and 1990s, decreased considerably between the 1990s and 2000s, and declined in a fluctuating manner between 2009 and 2018 [4]. Before the 1980s, the parent breeding cohorts of *P. gravieri* were caught by set-net and small-scale shrimp trawling in spring and summer [4]. From the 1980s, coastal fishermen in the East China Sea region fished using beam shrimp trawling [4]. Since then, with increasing shrimp trawler horsepower, the shrimp fishing ground area has expanded eastwards, with the exploitation of their overwintering and feeding groups distributed between 60 m and 100 m [4].

However, information on the migration route of *P. gravieri*, and its seasonal spatial distribution characteristics related to environmental factors, is fragmented. Such data limitations hinder our understanding of the biology and ecology of this species, and the performance of sustainable effective fisheries management measures based on total allowable catch (TAC). Moreover, climate change may influence the spatial distribution patterns of marine species via water temperature and salinity alterations [8]. In recent decades, sea bottom temperatures in the southern Bohai Sea have increased on average by 0.013 °C annually [9]. Sea bottom salinity, which was relatively stable at 28.7‰ between the 1950s and 1980s, increased to 30‰ at the beginning of the 2000s, increasing annually by 0.105‰ [10]. Currently, the species distribution model is widely used for determining variations in the habitat distribution patterns of marine organisms, including shrimp, and for predicting climate change impacts [11].

In this study, we describe the seasonal spatial distribution patterns, biomass, and abundance of *P. gravieri* in relation to environmental variables including depth, water temperature, and salinity, in order to identify its potential migration route across the seasons. We also employ the spatial distribution model to predict variations in its habitat under Shared Socioeconomic Pathway (SSP) climate change scenarios (SSP1-2.6, SSP2-4.5, SSP3-7.0, and SSP5-8.5) and across seasons. Our findings can help increase understanding of the distribution pattern and migration route of *P. gravieri*, and thus facilitate climate-induced sustainable fishery strategies and management according to TAC evaluations.

## 2. Materials and Methods

### 2.1. Geographical Characteristics of the Study Region and Surveying Procedures

The southern Yellow and East China Seas of China are part of the western continental marginal seas of the North Pacific. In a relatively short ~10,000-year earth history, several changes have occurred in this region, including sea–land changes, emergence of cold water masses, warm current expansion and decline, and evolution of coastal currents varying with the flow path of the Yangtze River (only occurring in the last 5000–9000 years). The main ocean currents in the Yellow and East China Seas are the Kuroshio warm and coastal cold currents, both of which are formed under the influence of cyclone circulation [12]. Coastal currents comprise the Huanghai and Donghai coastal currents, the seasonal variations in which are impacted by the monsoon and continental runoff [13]. The Taiwan warm current, as a branch of the Kuroshio, carries the warm subtropical water mass flowing into the East China Sea, which converges with coastal currents flowing southward at 30° N, and sinks to a 5 m depth to continuously flow northward. The Huanghai warm current, as a branch of the Kuroshio, with a large current volume, flows from south to north along the central axis of the Yellow Sea. This import causes higher sea surface water temperatures in the middle part of Yellow Sea compared to the coastal areas.

Moreover, in summer, the northern East China Sea is influenced by the Yangtze River diluted and Donghai–Huanghai mixed water masses. Historically, the Yangtze River diluted water mass extends to the west of Jeju Island, owing to the existence of a high-density water mass under the thermal layer south of Jeju Island, the outer current of which is expanding southeast [14]. In winter, the area is controlled by the Donghai–Huanghai mixed and East China Sea surface water masses. Due to the large reduction in the flow volume of the Yangtze River diluted water mass, sea surface salinity can increase to more than 30‰ ~100 km away from the east of the Yangtze River mouth. Because of the influence of northerly wind control and Kuroshio intrusion, the diluted water mass only expands southward in a narrow zone along the coast [15]. The Donghai-Huanghai mixed water mass controls half of the northern East China Sea owing to weak Yangtze River runoff, while the other half is controlled by the East China Sea surface water mass [15].

**Figure 1:**
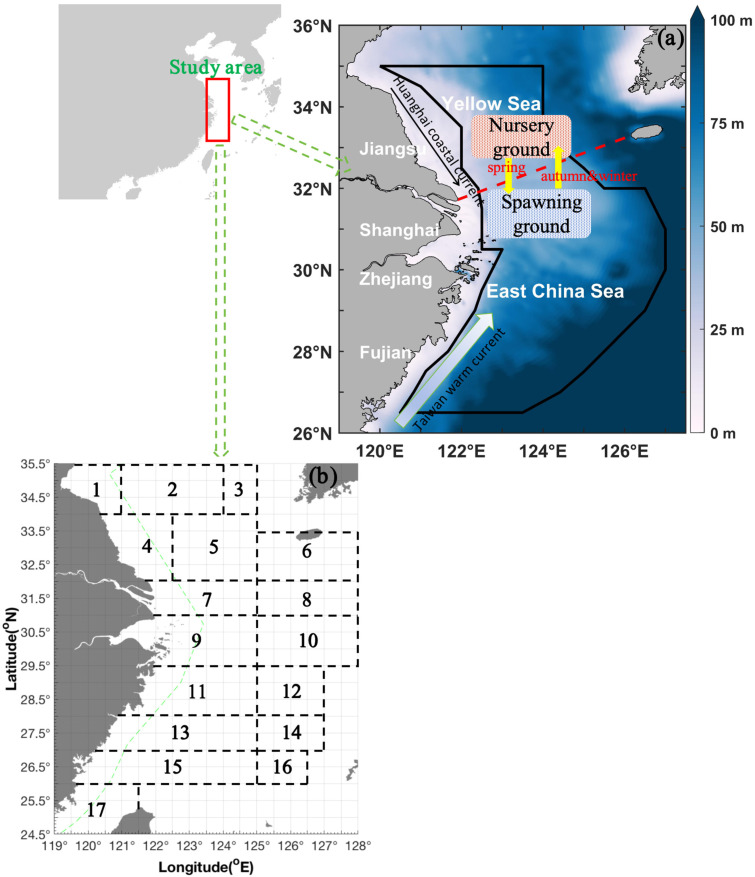
(**a**) Map of the study area (26.50–35.00° N, 120.00–127.00° E), denoted by a dark solid line, in the East China Sea region, including the southern Yellow and East China Seas. The color bar denotes the depth range from 0 to 100 m. The red dashed line indicates the boundary between the Yellow and East China Seas. (**b**) The black boxes and numbers represent the following fishing grounds: (1) Haizhou Bay, (2) Lianqingshi, (3) Liandong, (4) Lvsi, (5) Dasha, (6) Shawai, (7) Yangtze River mouth, (8) Jiangwai, (9) Zhoushan, (10) Zhouwai, (11) Yushan, (12) Yuwai, (13) Wentai, (14) Wenwai, (15) Mindong, (16) Minwai, and (17) Minzhong. The green dashed line indicates line of closed fishing area for bottom trawl fishery by motorboat.

We performed independent bottom trawling surveys in the southern Yellow and East China Seas (also called the East China Sea region) during 2018 and 2019 [16]. A map of the study area is presented in Figure 1. The surveys used a trawl net (Yongchang, Shengsi, China) with a 20 mm cod end mesh size and a height of 10–15 m, which was towed by fisheries research vessels in autumn (2–11 November 2018: 4601.15 g∙h^−1^ [50.63%] of total catch per unit effort by weight [CPUE_w_] and 3682.41 [51.51%] ind∙h^−1^ of total catch per unit effort by number [CPUE_n_]), winter (4–27 January 2019: 2897.08 [31.88%] g∙h^−1^ of total CPUE_w_ and 1993.78 ind∙h^−1^ [27.89%] of total CPUE_n_), spring (22 April–10 May 2019: 1555.46 g∙h^−1^ [17.11%] of total CPUE_w_ and 1453.19 ind∙h^−1^ [20.33%] of total CPUE_n_), and summer (13 August–27 September 2019: 34.91 g∙h^−1^ [0.38%] of total CPUE_w_ and 19 ind∙h^−1^ [0.27%] of total CPUE_n_). This indicated that the total CPUE_w_ and CPUE_n_ were ranked seasonally in the order of autumn > winter > spring >> summer. The survey stations were determined using a sampling grid with dimensions of 30 min of latitude and 30 min of longitude (30′ × 30′). The average trawl speed was 3 knots, and all tows were conducted for approximately 1 h at each station using a trawl net with a headline of 72.24 m and a groundline of 82.44 m. In total, 519 valid tows were included in this study (127 stations in autumn, 111 stations in winter, 141 stations in spring, and 140 stations in summer).

The growth equations were W_♀_ = 9.32 × 10^−6^ × (*L*)^3.1617^ and W_♂_ = 1.75 × 10^−5^ × (*L*)^2.9906^ [4]; and W_♀_ = 6.6415 × 10^−6^ × (*L*)^3.2094^, and W_♂_ = 2.3727 × 10^−5^ × (*L*)^2.8686^ [17]. Adult females were larger and grew faster than males [18].

The catches were analyzed in the laboratory for species identification and occurrence at each station. The total sample for each station was counted and weighed to the nearest 0.10 g of wet weight, and the catch density of *P. gravieri* was calculated as biomass density per unit of sampling time (g∙h^−1^) and density per unit of sampling time (ind∙h^−1^). The average individual weight (AIW) at each station was defined as the CPUE_w_ divided by the CPUE_n_. Environmental variables, including water depth, water temperature, and salinity were measured at each station using a conductivity-temperature-depth profiler (SBE-19; SeaBird-Scientific, Bellevue, WA, USA) [19].

### 2.2. Ensemble Model and Selection of Environmental Variables

Ottersen et al. (2010) suggested that the oceanographic parameters such as SST, SBT, SSS, and SBS, are important factors determining ocean circulation patterns, vertical mixing, availability of nutrients, and subsequent marine ecosystem primary production, which appear to be the leading indicators and important drivers of marine fishery resource fluctuations [20]. We used ensemble models to describe and predict the relationship between *P. gravieri* and environmental variables. The following 10 algorithms were utilized to predict the habitat distribution of *P. gravieri*: artificial neural network, classification tree analysis, flexible discriminant analysis, generalized additive model, generalized boosting model, generalized linear model, multiple adaptive regression splines, random forest, surface range envelope, and extreme gradient boosting training.

We used the ‘biomod2’ package 4.3-4 (https://biomodhub.github.io/biomod2/, accessed on 2 August 2025) in the ensemble species distribution modeling platform. To run the model, a random 80:20 ratio split was applied for training and testing data, respectively, to construct the 10 algorithms using the random cross-validation method [21]. We used the mean survey data over 4 months to produce an annual model, and applied different seasonal data to produce seasonal models. All data used in the models were obtained from the surveys conducted in this study. Future climate data were obtained from the Coupled Model Intercomparison Project Phase 6. Environmental data such as sea surface temperature (SST), sea bottom temperature (SBT), sea surface salinity (SSS), and sea bottom salinity (SBS) were obtained from the website Bio-ORACLE: marine data layers for ecological modeling (https://bio-oracle.org/index.php, accessed on 31 July 2025). Four SSP scenarios (SSP1–2.6, SSP2–4.5, SSP3–7.0, and SSP5–8.5) for the mid-term 2040–2050 (the 2050s) and long-term 2090–2100 (the 2090s) were used in this study.

## 3. Results and Discussion

### 3.1. Seasonal Variations in Environmental Factors

Seasonal ranges of environmental factors in the study area from autumn 2018 to summer 2019 are presented in Table 1. From summer to winter, SST decreased, while from winter to summer, it continuously increased (Table 1). The SST and SSS lower limit was 8 °C and 30‰, respectively (Table 1). It predominately inhabits coastal waters of 8–25 °C and 25–34‰ in the East China Sea [1], with the shortest molting period and fastest growth at water temperatures of 25 °C [22]. Other research showed that *P. gravieri* inhabits water temperatures of 5–25 °C and salinities of 25–34‰ [3]. Shi et al. (2022) found that acute high temperature (~30 °C) significantly reduced the metabolic capacity of the species but enhanced its immune capacity, which could be an emergency metabolic compensation technique to resist stress [23]. Shi et al. (2023) found that the response of the species to salinity change differed between hepatopancreas and gills, and the ion transport-related genes were mainly expressed in the gills [24]. The development of *P. gravieri* larvae from the zoea 1 to post-larval stage, takes 45 days at 22 °C, with an average daily growth rate of larvae of 0.0195 mm and incubation period of 10–14 days [25].

The SBT upper limits were ranked in the following order: summer > autumn > winter and spring, with a minimum value of 8 °C (Table 1). In spring, summer, and autumn, the SBS lower limit was 30.56–30.95‰, while in winter, it was 31.67‰, indicating that a lower salinity occurred in its habitat between spring and autumn, and that the population inhabited offshore area with higher salinity in winter (Table 1). The depth range values were similar between summer and autumn, and between spring and winter (Table 1).

**Figure 2:**
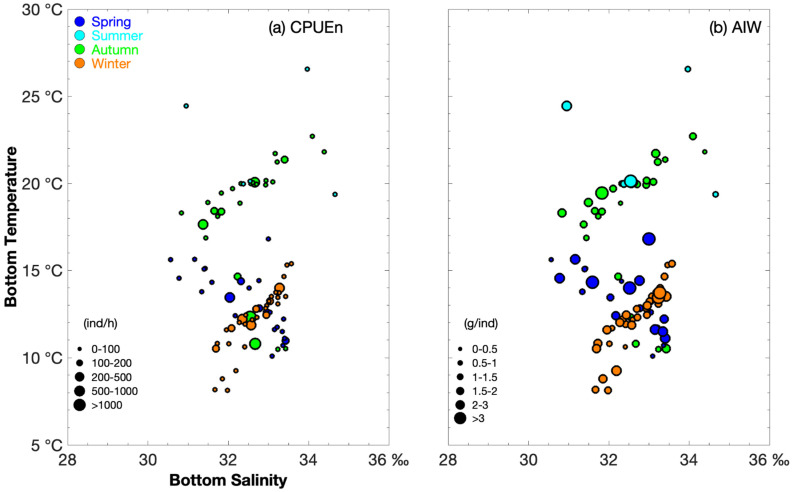
Relationship between salinity (‰) and temperature (°C) for *Palaemon gravieri* catch per unit effort by number (CPUE_n_) classified into groups (0–100, 100–200, 200–500, 500–1000, and >1000 ind·h^−1^) and average individual weight (AIW) sizes classified into groups (0–0.5, 0.5–1, 1–1.5, 1.5–2, 2–3, and >3 g·ind^−1^). The data for spring, summer, autumn, and winter are denoted by solid blue, light blue, green, and brown-red circles, respectively. (**a**) Sea bottom temperature vs. sea bottom salinity for CPUE_n_; (**b**) Sea bottom temperature vs. sea bottom salinity for AIW.

Figure 2 shows the relationship between SBS and SBT for CPUE_n_ and AIW. In spring, the highest *P. gravieri* abundance (CPUE_n_ > 100 ind h^−1^) was recorded at a SBT of 12.6–14 °C and a SBS of 32–33‰ (Figure 2). In autumn, the highest abundances occurred at 10.8–12.3 °C and 33‰ (CPUE_n_ > 500 ind h^−1^) and at 17.6–21.4 °C and 31.4–33.4‰ (CPUE_n_ 100–500 ind h^−1^) (Figure 2). In winter, the highest abundance (CPUE_n_ > 100 ind h^−1^) was recorded at 10.5–14 °C and 31.7–33.3‰ (Figure 2).

The groups with an AIW > 2 g·ind^−1^ were found at a SBT of 11.1–16.8 °C and a SBS of 30.8–33.4‰, while those with an AIW < 1 g·ind^−1^ occurred at 10.1–15.6 °C and 30.6–33.4‰ in the spring (Figure 2). In summer, groups with an AIW > 2 g·ind^−1^ were recorded at 20.1–24.5 °C and 31–32.5‰, while those < 1 g·ind^−1^ were found at 19.4–26.6 °C and 34–34.7‰ (Figure 2). In autumn, groups with an AIW > 1.5 g·ind^−1^ occurred at 18.3–21.7 °C and 30.8–33.2‰, and those with an AIW < 1 g·ind^−1^ at 16.9–21.8 °C and 31.4–34.4‰ (Figure 2). In winter, > 2 g·ind^−1^ groups were recorded at 13–14 °C and 33‰, and <1 g·ind^−1^ groups at 10.6–15.3 °C and 32–33.5‰ (Figure 2).

Table 2 shows the range in SST, SSS, SBT, SBS, and depth in the fishing grounds of Dasha and the Yangtze River mouth across the seasons (Figure 1 and Table 2).

### 3.2. Seasonal Spatial Distribution Characteristics and Patterns

**Figure 3:**
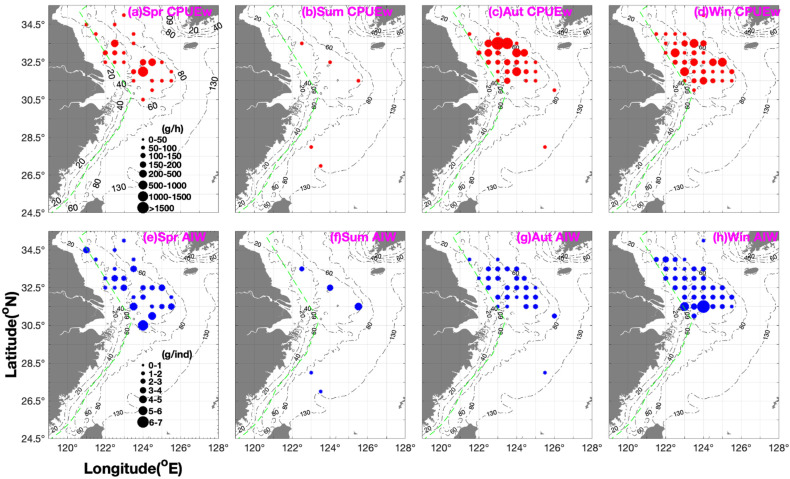
Seasonal distribution patterns of *Palaemon gravieri* catch per unit effort by weight (CPUE_w_; g·h^−1^). CPUE_w_ is shown in red (grouped into 0–50, 50–100, 100–150, 150–200, 200–500, 500–1000, 1000–1500, and >1500 g·h^−1^) and average individual weight (AIW; g·ind^−1^) is shown in blue (grouped into 0–1, 1–2, 2–3, 3–4, 4–5, 5–6, and 6–7 g·ind^−1^). (**a**–**d**) CPUE_w_ in (**a**) spring, (**b**) summer, (**c**) autumn, (**d**) winter; (**e**–**h**) AIW in (**e**) spring, (**f**) summer, (**g**) autumn, and (**h**) winter. The green dashed line indicates line of closed fishing area for bottom trawl fishery by motorboat.

Figure 3 and Table 3 present the seasonal spatial distribution patterns of *P. gravieri* in the southern Yellow and East China Seas. In spring, the mean CPUE_w_ of the fishing grounds was ranked in the order of Yangtze River mouth > Dasha and Lvsi >> Haizhou Bay, while for mean CPUE_n_, it was Lvsi > Dasha and Yangtze River mouth >> Haizhou Bay (Figure 3 and Table 3). AIW was ranked in the order of Yangtze River mouth > Dasha > Lvsi > Haizhou Bay (Figure 3 and Table 3). Yi (2012) recorded a large body length of 50–60 mm in the Yangtze River mouth from March to April [26]. This AIW reduction with increasing latitude indicates the possibility of a spawning ground in the Yangtze River mouth. The longitudinal ranking of CPUE_w_ and CPUE_n_ was 124° E–124.5° E > 121° E–122.5° E > 123° E–123.5° E > 125° E–125.5° E, while that of AIW was 124° E–124.5° E > 123° E–123.5° E and 125° E–125.5° E > 121° E–122.5° E, indicating the existence of smaller individuals in the inshore areas (Figure 3 and Table 3).

In summer (August–September), the mean CPUE_w_ and CPUE_n_ of the fishing grounds considerably decreased in the order of Yangtze River mouth > Dasha and Wentai. AIW was ranked in the order of Yangtze River mouth > Dasha > Wentai, with larger individuals in the southern Yellow and northern East China Seas, and smaller individuals in the southern East China Sea. The longitudinal ranking of AIW was 125.5° E > 124° E > 122.5° E > 123° E–123.5° E (Figure 3 and Table 3).

In autumn, fishing ground mean CPUE_w_ and CPUE_n_ were ranked in the order of Dasha > Lvsi > Yangtze River mouth, while mean AIW was in the order of Dasha > Lvsi and Yangtze River mouth, indicating the possibility of an overwintering nursery ground in the Dasha fishing ground. The longitudinal ranking of CPUE_w_, CPUE_n_, and AIW was 123° E–123.5° E > 124° E–124.5° E > 121.5° E–122.5° E > 125° E–126° E, indicating a concentrated area of recruitment at 123° E–124° E and a recruitment population in the offshore areas of 125° E–126° E (Figure 3 and Table 3).

In winter, fishing ground CPUE_w_ and CPUE_n_ were ranked in the order of Dasha > Yangtze River mouth > Lvsi, while mean AIW was in the order of Yangtze River mouth > Lvsi and Dasha. The longitudinal ranking of CPUE_w_ and CPUE_n_ was 123° E–123.5° E > 124° E–124.5° E > 125° E–125.5° E > 121.5° E–122.5° E, while that of AIW was 124° E–124.5° E > 123° E–123.5° E > 121.5° E–122.5° E > 125° E–125.5° E (Figure 3 and Table 3).

Generally, *P. gravieri* in China is considered to be mainly distributed in the fishing grounds of Lvsi, Yangtze River mouth, and Zhebei in the southern Yellow and northern East China Seas, with its CPUE_w_ longitudinally ranked in the order of 31° E–33° E > 28° E–31° E [1]. It is concentrated in the intersection area of the Yangtze River diluted and offshore high-salinity waters [4]. Its biomass and abundance distribution is strongly impacted by Yangtze River runoff [15].

Table 4 shows the mean seasonal CPUE_w_, CPUE_n_, and AIW of *P. gravieri* from autumn 2018 to summer 2019. According to season, CPUE_w_ was ranked in the order of autumn > winter > spring > summer; CPUE_n_ was ranked autumn > winter and spring; and AIW was ranked summer > spring > autumn > winter (Table 4), highlighting the importance of the summer fishing moratorium in China (1 May–31 August annually). Other research has shown that the highest biomass and abundance of *P. gravieri* occurs in autumn and winter [1]. In the East China Sea region, catches were ranked seasonally in the order of spring >> autumn > summer and winter, with a CPUE_w_ of 3110, 2640, 1610, 1990, 790, 1810, 2510, 810, 400, and 670 g·ind^−1^ from 2009 to 2018, respectively [4]. The seasonal ranking of the estimated potential total biomass was autumn (14,744.1 t) > winter (10,844.5 t) > spring (6081.5 t) and summer (6062.7 t) from 1998 to 1999 [4].

Additionally, there were two spawning cohorts in spring and autumn. Shrimp in the spring cohort grew to 35 mm between July and August, with rapid growth from August to October, and then grew to 45 to 55 mm in November to December, and to 55 to 70 mm in January to March. They reached sex maturity and reproduced between April and June [27]. Previous research also reported five stages of ovarian maturity: immature ovaries from September to November; first ovarian occurrence in December; first mature females appearing in January; increasing proportion of mature females from February until April; and first broods appearing in April [26]. The sex ratio of females to males is highest in March [26]. Shrimp in the autumn cohort reproduced from September to November and grew to 30 to 45 mm from November to December, with a rapid growth stage in spring, attaining 55 to 70 mm between August and October [27]. Large-sized individuals (~50 to 60 mm body length) were found in March to June, comprising the spawning groups, and juveniles were recorded between July and November, comprising the feeding groups [5]. In the warm-temperate waters of southern Korea, the breeding period of the species begins in March, peaking (ovigerous females) in May, and then ends by August, and is constrained by temperature and release of larvae coinciding with plankton blooms [28].

### 3.3. Habitat Predictions Across Seasons and Under Different Climate Scenarios

**Figure 4:**
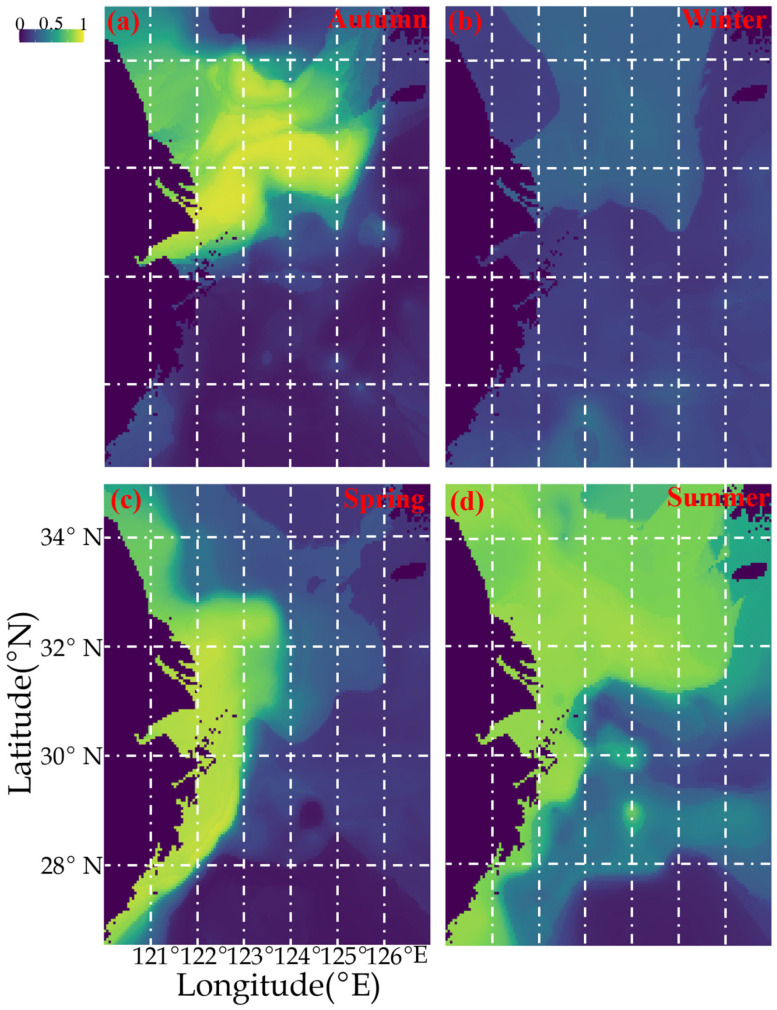
Current seasonal spatial distribution patterns of *Palaemon gravieri* from autumn to summer (**a**–**d**) in the study area based on data collected from 2018–2019.

Figure 4 shows the current estimated seasonal spatial distribution pattern of *P. gravieri* (based on 2018–2019 data). In spring, suitable habitat was concentrated in the Yangtze River mouth fishing ground and inshore areas of the southern Yellow and East China Seas (Figure 4). In summer, suitable habitat included the southern Yellow Sea and inshore areas of the East China Sea (Figure 4). In autumn, *P. gravieri* was concentrated in the southern Yellow and northern East China Seas, while in winter, it occurred in the offshore areas of the southern Yellow and northern East China Seas (Figure 4).

**Figure 5:**
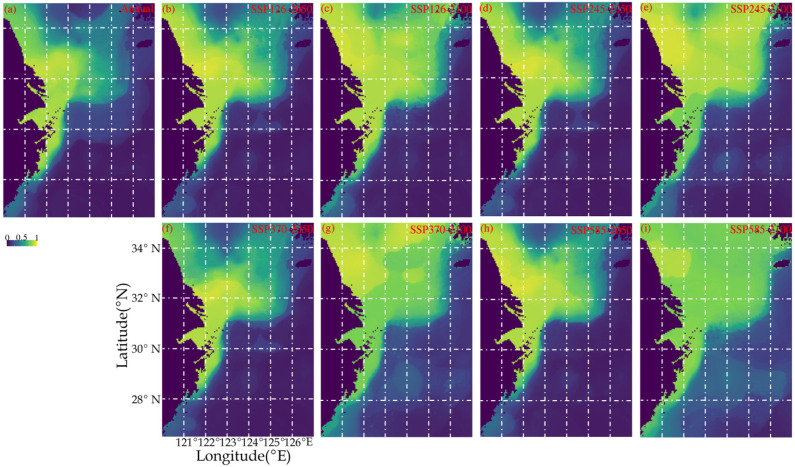
Predicted spatial habitat distribution patterns of *Palaemon gravieri* under difference scenarios (**a**) annual mean habitat in 2018–2019, (**b**) SSP126 in 2050, (**c**) SSP126 in 2100, (**d**) SSP245 in 2050, (**e**) SSP245 in 2100, (**f**) SSP376 in 2050, (**g**) SSP370 in 2100, (**h**) SSP585 in 2050, and (**i**) SSP585 in 2100.

Figure 5 and Table 5 provide the percentage gain and loss of suitable habitat area under different climate scenarios. *Palaemon gravieri* was mainly concentrated northwards of 28.5° N (Figure 5). Under the current scenario, it was mainly concentrated in the inshore areas of the southern Yellow and northern East China Seas (Figure 5). This was similar under SSP1-2.6, SSP2-4.5, and SSP5-8.5 scenarios in 2050, but with a greater offshore enlarged tendency (Figure 5). Under SSP1-2.6, SSP2-4.5, SSP3-7.0, and SSP5-8.5 scenarios in 2100, *P. gravieri* was predominately concentrated in areas of the southern Yellow and northern East China Seas, and in the inshore areas of the East China Sea (Figure 5). Under the SSP3-7.0 scenario in 2050, the suitable habitat areas included the fishing grounds of Lvsi and Haizhou Bay, the area controlled by Yangtze River diluted water, and the inshore water areas of the northern East China Sea (Figure 5).

Regarding the percentage habitat area loss, most scenarios were associated with loss of <5% in the 2050s and an almost 0% loss in the 2100s (Table 5). In terms of the percentage habitat area gain, the scenarios were ranked in the order of SSP585-2100 > SSP370-2100 > SSP126-2100 and SSP245-2100 > scenarios in 2050s, showing greater percentage area gain in the 2100s compared to in the 2050s (Table 5). Regarding overall habitat (percentage area gain minus loss), all scenarios had positive values, with SSP585-2100 and SSP126-2050 being the most beneficial and detrimental scenarios, respectively (Table 5).

### 3.4. Environmental Adaptive Sustainable Fisheries Management Strategies

Natural resource management schemes are basic tools for the sustainable utilization of commercial fisheries, including trawling fisheries. Knowledge of different biological aspects of the life history traits, including seasonal spatial distribution pattern and migration, of the species population or cohorts can help in the temporal modeling of group dynamic variations and prediction of potential future distribution patterns under different climate scenarios. The *P. gravieri* distribution patterns, relationships with environmental variables, and predictions under climate scenarios revealed in this study are vital for future fisheries management, such as controlling the grid net mesh to >50 mm in beam shrimp trawling, setting the total allowable catch in Dasha and the Yangtze River mouth fishing grounds, and protecting the spawning cohorts from March to April each year. However, this study has some limitations. Specifically, the methods used have a potential risk of overfitting when predicting future distributions of the species under different climate scenarios [29].

## 4. Conclusions

Over the previous ~30 years (1995–2025), fisheries management practices, overfishing, and climate change have been the main focus of coastal fishermen and governmental fisheries managers in the East China Sea region. First, they need to understand the role of the summer fishing moratorium in managing various fisheries resources, and present new management strategies such as TAC. Second, after 30 years of overfishing practices, it is vital to understand the current seasonal spatial distribution patterns of species under climate scenarios. This study demonstrates that, in spring, the majority of the *P. gravieri* population migrates from the offshore areas in the Dasha fishing ground to the inshore areas of the Yangtze River mouth fishing ground for spawning. In autumn, recruitment migration occurs from the Yangtze River mouth fishing ground to the Dasha fishing ground, comprising overwintering and nursery grounds. This results in seasonal migration from the east to the west, and from the south to the north. These findings can benefit future TAC fisheries management.

## Figures and Tables

**Table 1:** Seasonal ranges and mean values of environmental factors (SST, SSS, SBT, SBS, and depth) in the study area from autumn 2018 to summer 2019.

Factor	Spring	Summer	Autumn	Winter
	Value Range			
SST (°C)	12.61–17.2	25.2–28.75	16.97–23.72	8.09–15.36
SSS (‰)	30.51–33.49	29.89–34.15	30.53–34.24	31.6–33.6
SBT (°C)	10.1–16.81	19.38–26.56	10.47–22.7	8.14–15.37
SBS (‰)	30.56–33.42	30.95–34.66	30.84–34.39	31.67–33.57
Depth (m)	19–73	31–113	14–105	11–78
	Mean value			
SST (°C)	14.65	26.83	19.89	12.08
SSS (‰)	31.97	32.03	32.16	32.61
SBT (°C)	13.24	22.1	18.3	12.25
SBS (‰)	32.46	32.9	32.53	32.67
Depth (m)	44.04	62	44.57	41.09

Abbreviations: SST, sea surface temperature; SBT, sea bottom temperature; SSS, sea surface salinity; SBS, sea bottom salinity.

**Table 2:** Range of SST, SSS, SBT, SBS, and depth in the fishing grounds of Dasha and the Yangtze River mouth across the seasons.

Seasons	Fishing Grounds	SST (°C)	SSS (‰)	SBT (°C)	SBS (‰)	Depth (m)
Spring	Dasha	12.6–16.7	30.6–33.5	10.9–15.6	31.2–33.4	32–70
	Yangtze River mouth	13.2–17.2	30.8–32.9	11.6–16.8	31.3–33.4	39–66
Summer	Dasha	25.2–27.6	29.9–31.6	20–24.5	31–32.4	31–39
	Yangtze River mouth	28.8	30.5	20.1	32.5	52
Autumn	Dasha	18–21.2	30.5–32.6	10.5–20	30.84–33.43	28–65
	Yangtze River mouth	19.9–22.7	32.2–34	19.9–22.7	32.31–34.09	35–69
Winter	Dasha	10.7–13.6	31.6–33.1	10.8–14	31.73–33.28	29–65
	Yangtze River mouth	11.7–15.4	32.4–33.6	11.9–15.4	32.57–33.57	35–60

**Table 3:** Mean and total values of catch per unit effort by weight (CPUE_w_; g·h^−1^), percentage of CPUE_w_, catch per unit effort by number (CPUE_n_; ind·h^−1^), percentage of CPUE_n_, average individual weight (AIW; g·ind^−1^), and percentage of AIW in different fishing grounds according to season.

	Mean Value					Total Value					
	B	B%	N	N%	AIW	B	B%	N	N%	AIW	AIW%
	Spring										
(1)	7.3	3.42%	24.8	11.31%	1.0	21.9	1.41%	74.5	5.13%	3.1	7.61%
(4)	64.1	30.10%	88.5	40.34%	0.8	384.9	24.74%	531.2	36.56%	5.0	12.14%
(5)	63.1	29.59%	53.9	24.54%	1.8	441.4	28.38%	377.0	25.94%	12.9	31.45%
(7)	78.6	36.88%	52.3	23.81%	2.2	707.3	45.47%	470.4	32.37%	20.0	48.80%
	Summer										
(5)	5.7	21.94%	2.3	19.54%	2.0	11.4	63.60%	4.7	33.50%	4.0	74.08%
(7)	17.0	65.50%	5.0	41.67%	3.4						
(13)	3.3	12.56%	4.7	38.79%	0.7	6.5	36.40%	9.3	66.50%	1.4	25.92%
	Autumn										
(4)	104.0	22.88%	84.0	23.01%	1.1	519.8	11.30%	420.2	11.42%	5.7	12.70%
(5)	287.7	63.31%	222.6	60.94%	2.3	3452.3	75.06%	2671.6	72.63%	27.9	62.04%
(7)	62.7	13.81%	58.7	16.06%	1.1	627.5	13.64%	586.6	15.95%	11.4	25.26%
	Winter										
(4)	45.8	18.33%	31.4	18.16%	1.4	366.6	12.66%	251.0	12.60%	11.1	20.84%
(5)	124.8	49.93%	95.8	55.48%	1.4	1498.2	51.72%	1150.0	57.71%	16.4	30.73%
(7)	79.4	31.75%	45.5	26.35%	2.0	1031.9	35.62%	591.8	29.70%	25.8	48.42%

N.B.: fishing grounds of (1) Haizhou Bay, (4) Lvsi, (5) Dasha, (7) Yangtze River mouth, and (13) Wentai (see Figure 1).

**Table 4:** Seasonal data for catch per unit effort by weight (CPUE_w_; g·h^−1^), catch per unit effort by number (CPUE_n_; ind·h^−1^), and average individual weight (AIW; g·ind^−1^) from autumn 2018 to summer 2019.

Factor	Spring	Summer	Autumn	Winter
Mean CPUE_w_ at collection stations (g·h^−1^)	62.22	6.98	164.33	85.21
Value range of CPUE_w_ (g·h^−1^)	0.31–528.82	0.7–17	1.38–1510.4	0.4–465.6
Mean CPUE_n_ at collection stations (ind·h^−1^)	58.13	3.8	131.51	58.64
Value range of CPUE_n_ (ind·h^−1^)	1.03–362.04	1–8.31	3.21–1280	1–304
Mean AIW (g·ind^−1^)	1.64	1.76	1.16	1.58
Value range of AIW (g·ind^−1^)	0.15–5.02	0.7–3.4	0.3–1.92	0.4–6.4

Abbreviations: CPUE_w_, catch per unit effort by weight; CPUE_n_, catch per unit effort by number; AIW, average individual weight.

**Table 5:** Percentage habitat loss, gain, and overall habitat (gain minus loss) for *Palaemon gravieri* under various climate scenarios (SSP126-2050, SSP126-2100, SSP245-2050, SSP245-2100, SSP370-2050, SSP370-2100, SSP585-2050, and SSP585-2100).

Case	Loss%	Gain%	Gain%−Loss%
SSP126–2050	−6.73%	8%	1.27%
SSP126–2100	−0.44%	16.46%	16.02%
SSP245–2050	−5.74%	7.78%	2.04%
SSP245–2100	−0.62%	19.34%	18.72%
SSP370–2050	−4.66%	7.43%	2.77%
SSP370–2100	−0.08%	27.14%	27.06%
SSP585–2050	−3.83%	8.14%	4.31%
SSP585–2100	0%	59.02%	59.02%

## Data Availability

The original contributions presented in this study are included in the article. Further inquiries can be directed to the corresponding author(s).
